# Do periodic arm movements during sleep exist in healthy subjects? A polysomnographic study

**DOI:** 10.1016/j.sleep.2014.05.014

**Published:** 2014-09

**Authors:** David Gabelia, Thomas Mitterling, Birgit Högl, Gregor K. Wenning, Birgit Frauscher

**Affiliations:** Department of Neurology, Innsbruck Medical University, Innsbruck, Austria

**Keywords:** Periodic arm movements, Sleep, Healthy subjects, Polysomnography, AASM 2.0

## Abstract

**Background:**

Despite several polysomnographic studies on periodic leg movements (PLM) in healthy sleep, data on the prevalence and characteristics of periodic arm movements (PAM) in normal subjects are lacking. We aimed to investigate PAM and their association with PLM during wakefulness and sleep in healthy subjects.

**Methods:**

Ninety-one participants underwent video-polysomnography according to American Academy of Sleep Medicine 2007 criteria. In addition to standard electromyographic registration, data for both flexor digitorum superficialis muscles were recorded.

**Results:**

Sixty-two subjects (68.1%) had a PAM index during wakefulness >5/h (median PAM index during wakefulness, 8.8/h; range, 0–77). Seven subjects (7.7%) had a PAM index >5/h during sleep (median PAM index during sleep, 0.7/h; range, 0–47.4). In 14% of cases, PAM during wakefulness were coincident with PLM during wakefulness. During sleep, this coincidence was not evident. The correlation between PAM and PLM was weak to moderate (during wakefulness: Spearman's *ρ* = 0.576, *P* < 0.001; during sleep: Spearman's *ρ* = 0.222, *P* = 0.036).

**Conclusion:**

In healthy subjects, PAM occur predominantly during wakefulness with no apparent true periodicity. In contrast to classical PLM, some PAM may not present a true periodic phenomenon, but rather random voluntary movements meeting the wide range of periodicity criteria for PLM.

## 1 Introduction

Despite multiple studies on periodic leg movements (PLM) in wakefulness (PLMW) and sleep (PLMS) [1], data on periodic arm movements (PAM) in wakefulness (PAMW) and sleep (PAMS) are scarce, and have been reported only in restless legs syndrome (RLS) or other neurological disorders [2] , [3] , [4] , [5] , [6] , [7] , [8] , [9] . So far, the literature on PAM comprises 29 subjects (26 patients with RLS, one patient with multiple sclerosis, one patient with corticobasal degeneration, one patient with cervical spondylosis C4/C5) [2] , [3] , [4] , [5] , [6] , [7] , [8] , [9] . Of these 29 subjects, 22 RLS patients were reported in one case series [9]. The authors found that ~70% of the investigated RLS subjects had a PAMW index >5/h, whereas 14% of cases also showed a PAMS >5/h. Approximately 40% of PLMW were accompanied by PAMW. Based on these findings, the authors concluded that PAM are more frequent than generally expected [9].

As PLM prevalence increases in healthy subjects with advanced age [10] , [11] , [12] , [13] , [14] , [15] , [16] , we were interested in investigating PAM during wakefulness and sleep in a sample of healthy subjects over the lifespan. We hypothesized that PAM are present in physiological sleep and that their frequency might increase with age. Therefore the aim of the present study was to prospectively investigate PAM during wakefulness and sleep in healthy subjects over the lifespan, and to assess the association between PAM and PLM in physiological sleep.

## 2 Methods

### 2.1 Study sample and polysomnographic registration

Ninety-one healthy subjects (55 men, 36 women; median age, 42 years; range, 19–77) were selected from a sample of healthy sleepers who had undergone video-polysomnography (PSG) at the tertiary sleep disorder referral center of Innsbruck Medical University between August and November 2011 for a research project on motor activity during healthy sleep [16]. All participating volunteers underwent a two-step screening process (telephone interview, personal investigation by a physician trained in sleep medicine) to exclude a relevant sleep, neurological, psychiatric or internal comorbidity as well as any use of central nervous system medication (for detailed methodology see [16]).

Video-PSG was performed according to the American Academy of Sleep Medicine (AASM) 2007 guidelines. For electromyographic (EMG) recording, The SINBAR EMG montage was used (mentalis, flexor digitorum superficialis on the upper extremities, tibialis anterior on the lower extremities) [17].

The study was approved by the local ethics committee of Innsbruck Medical University. All patients gave written informed consent.

### 2.2 Derived measures

Sleep staging was performed according to AASM 2007. Arm movements (AM) and PAM as well as leg movements (LM) and PLM were scored according to the criteria of the World Association of Sleep Medicine [18]. We defined PAM and PLM to be coincident when both occurred at an interval of ≤0.5 s apart, irrespective of which one was first. To further characterize PAM and PLM, the periodicity index was calculated, defined as the number of LM occurring in a sequence of three intermovement intervals with a duration between 10 and 90 s divided by the total number of intermovement intervals exceeding 1 s [19].

### 2.3 Statistics

Statistical analysis was performed with IBM SPSS Statistics 20.0 for Windows. As data were not normally distributed, they were reported as median (range). For statistical analysis, non-parametric Mann–Whitney U-test in the case of two groups and Kruskal–Wallis test in the case of two or more groups were applied. To assess bivariate correlations of PAM and PLM variables, Spearman correlation coefficients were calculated. In order to investigate the influence of age on PAM and PLM parameters, participants were pooled in five age groups: subjects aged <30 years: 18 (eight women, 10 men); 30–39 years: 21 (10 female, 11 male); 40–49 years: 16 (12 female, four male); 50–59 years: 13 (eight female, five male); and subjects aged ≥60 years: 23 (17 female, six male). *P* < 0.05 was considered statistically significant. As this study was exploratory in nature, testing for multiple comparisons was not performed.

## 3 Results

### 3.1 Periodic arm movement and periodic leg movement indices

**Fig. 1:**
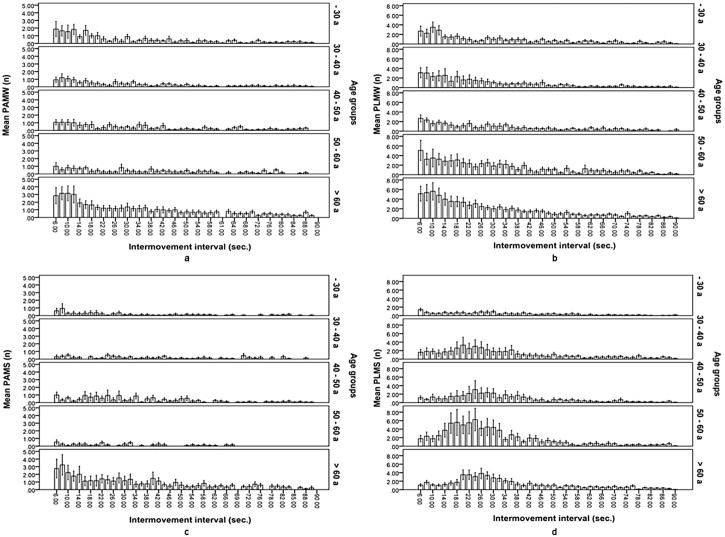
Intermovement interval graphs of periodic arm and leg movements: (a) periodic arm movements in wakefulness; (b) periodic leg movements in wakefulness; (c) periodic arm movements in sleep; (d) periodic leg movements in sleep.

Table 1 provides PAM and PLM indices for the total group as well as the five different age groups. PAMW were not uncommon, and 62 participants (68.1%) had a PAMW index >5/h. During sleep, the PAM index was considerably lower: only seven volunteers (7.7%) had a PAMS index >5/h. The PAM periodicity index was very low at 0.04 (range, 0–0.49). Compared with median PAMW and PAMS indices, median PLMW and PLMS indices were higher. The same was true for the number of subjects with PAMW and PAMS index >5/h: 75 subjects (82.4%) had a PLMW index >5/h, and 31 (34.1%) had a PLMS index >5/h. The PLM periodicity index was 0.28 (0–1). Figure 1 shows the intermovement interval graphs of PAM and PLM during both wakefulness and sleep.

**Table 1:** Comparison of characteristics of arm and leg movements.

Movements	Total	Age (years)					*P*-value
		<30	30–39	40–49	50–59	≥60	
Arms							
Total night AM index^a^	11.3 (4.1–66.8)	10.1 (6.1–28.1)	11.8 (4.2–59.1)	11.1 (4.4–24.1)	11.3 (6.9–16.6)	12.3 (4.4–65.8)	0.914^c^
Total night PAM index^a^	2.3 (0–51)	2.3 (0–19.5)	2.2 (0–38)	2.7 (0.6–14)	2.1 (0.5–5.6)	3.5 (0–51)	0.745^c^
PAMW index^a^	8.8 (0–77)	13.9 (0–77)	7.7 (0–31.8)	10 (1.4–54)	5.8 (0–28.3)	9.4 (0–64.6)	0.634^c^
PAMW >5/h^b^	62 (68.1%)	12 (66.7%)	15 (71.4%)	12 (75%)	9 (69.2%)	14 (60.9%)	0.789^d^
PAMS index^a^	0.7 (0–47.4)	0.6 (0–11.1)	0.5 (0–42.2)	0.8 (0–14.6)	0.6 (0–2.4)	1.2 (0–47.4)	0.667^c^
PAMS >5/h^b^	7 (7.7%)	1 (5.6%)	1 (4.8%)	1 (6.3%)	0 (0%)	4 (17.4%)	0.277^d^
PAM arousal index^a^	1.6 (0–7.3)	1.8 (0.3–3.9)	1.7 (0.3–4.3)	0.8 (0.1–3.6)	1.4 (0.2–3.2)	1.9 (0–7.3)	0.755^c^
Periodicity index^a^	0.04 (0–0.49)	0.03 (0–0.13)	0.03 (0–0.27)	0.05 (0–0.49)	0.05 (0–0.16)	0.07 (0–0.39)	0.459^c^
Legs							
Total night LM index^a^	17.7 (2.5–72)	14.3 (4.9–37.3)	16 (3.6–72)	16 (2.5–41)	27.2 (6.3–57.3)	24.9 (4.4–65.8)	0.014^c^
Total night PLM index^a^	8.3 (0–61.9)	4 (0–21.9)	8 (0–61.9)	5.6 (0.6–14)	14.7 (0.6–40.4)	15.4 (0–53)	0.026^c^
PLMW index^a^	26.7 (0–115)	25.9 (0–115)	28 (0–105.1)	24.4 (0–100.5)	25 (0–92)	29.2 (0–111.8)	0.616^c^
PLMW >5/h^b^	75 (82.4%)	13 (72.2%)	17 (81%)	13 (81.3%)	11 (84.6%)	21 (91.3%)	0.371^d^
PLMS index^a^	2.8 (0–60.9)	2.1 (0–12.1)	3.8 (0–60.9)	1.7 (0–31.6)	5.7 (0–41.6)	3.4 (0–34.3)	0.207^c^
PLMS >5/h^b^	31 (34.1%)	3 (16.7%)	8 (38.1%)	3 (18.8%)	7 (53.8%)	10 (43.5%)	0.181^d^
PLM arousal index^a^	0.5 (0–5.6)	0.1 (0–2.4)	0.2 (0–3.7)	0.3 (0–4.9)	0.6 (0–5.6)	1.1 (0–5.4)	0.039^c^
Periodicity index^a^	0.28 (0–1)	0.24 (0–0.13)	0.22 (0–0.27)	0.23 (0–0.80)	0.31 (0–1)	0.37 (0–1)	0.376^c^

AM, arm movements; PAM, periodic arm movements; PAMW, periodic arm movements in wakefulness; PAMS, periodic arm movements in sleep; LM, leg movements; PLM, periodic leg movements; PLMW, periodic leg movements in wakefulness; PLMS, periodic leg movements in sleep.

a Reported values are expressed as medians (ranges).

b Reported values are expressed as numbers of participants (percentages).

c Kruskal–Wallis test.

d χ^2^-Test.

**Fig. 2:**
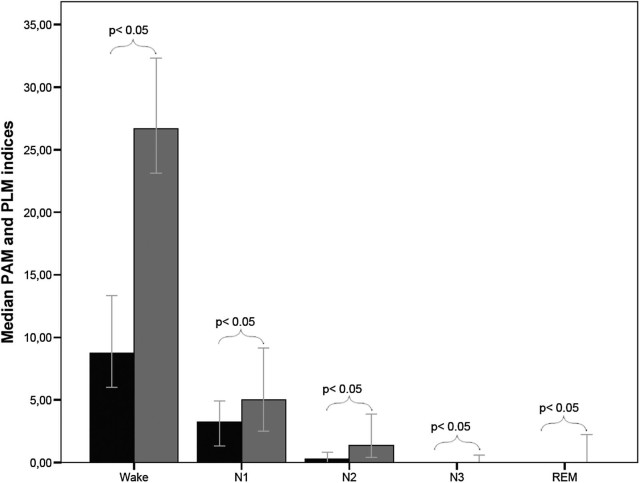
Distribution of periodic arm movement (PAM) and periodic leg movement (PLM) indices during wakefulness and sleep. Black bars: PAM indices; grey bars: PLM indices. Whiskers: 95% confidence intervals.

The sleep stage distribution showed highest PAM indices in N1 sleep with 3.2/h (0–78) followed by N2 sleep (median, 0.2/h; range, 0–55.8), N3 sleep (median, 0/h; range, 0–108.8) and REM sleep (median, 0/h; range, 0–43.5). This distribution was comparable with that of PLM, with PAM indices being lower for all sleep stages compared to PLM indices (Fig. 2).

### 3.2 Coincidence and correlation of PAM and PLM

PAMW coincided with PLMW in a median of 14.8% of cases (0–100%), whereas PAMS did not coincide with PLMS in the median of cases (0–88%). The correlation between PAM and PLM was moderate during wakefulness (*P* = 0.576, *P* < 0.001) and only weak during sleep (*ρ* = 0.222, *P* = 0.036).

### 3.3 Changes of PAM and PLM over the lifetime

There was no change of PAM variables over the different age groups (all *P* > 0.05, see Table 1). Concerning PLM variables, only the total PLM index increased with more advanced age (*P* < 0.05).

## 4 Discussion

This is the first study investigating PAM in a sample of healthy sleepers recruited from a representative sample of the general population. Our main finding is that PAM are not uncommon in the healthy population, but that their number is markedly lower compared to that of PLM. Moreover, PAM occur predominantly during wakefulness or light sleep with very low periodicity, and coincidence between PAM and PLM was low.

The findings demonstrate that PAM are a widespread motor phenomenon in the healthy population. Surprisingly, the percentage of subjects with PAMW index >5/h was identical to that reported for RLS [9]. Whether arm movements in RLS are more extended, or even visible, is a matter of speculation. In the normal subjects examined in this study, no obvious arm movements were visible in the synchronized video-recording during investigation of PAM EMG activations. As recording of upper arm muscles is not routinely performed, this observation might also explain why PAM are not more frequently described in the literature.

Our data showed that the coincidence between PAM and PLM was only low during wakefulness and absent during sleep. Moreover, the correlation between PAM and PLM was only weak to moderate. These results are in line with the first case description of PAM, which demonstrated a desynchronization between PAM and PLM. Based on this finding, the authors speculated on different generator sites of PAM and PLM [3]. Further evidence was raised from a different perspective by a study on the recruitment pattern of PLMS in RLS patients. The authors demonstrated that in PLM, the involvement of axial and upper limb muscles (biceps brachii and triceps brachii) was rather rare [20].

In our study, PAM were predominantly present during wakefulness and light sleep, and their periodicity was very low; therefore one might argue that at least some PAM were simply voluntary or comfort movements and hence should be classified as distinct from true PLM. This statement might be confusing at first glance, but one has to keep in mind that the definition of periodicity in PLM and PAM is based exclusively on the presence of an intermovement interval of between 5 and 90 s, not on a regular occurrence within this interval. The latter, however, is much better reflected by the periodicity index as proposed by Ferri et al. [19].

Studies on PLM in healthy controls showed that PLM increased with age [10]. This is also true for the present study, which demonstrated a positive association between LM and PLM indices over the different age groups. In contrast to this finding, PAM indices did not differ between the different age groups. At first glance, this finding is surprising, but it might point to a distinct etiology of PAM and PLM.

In this study, PAM were assessed in the flexor digitorum superficialis muscle, because it is the recommended muscle for the SINBAR montage for detection of rapid eye movement sleep behavior disorder [17], and is included in the routine polysomnographic montage in our laboratory. The usefulness of this muscle for the detection of PAM has not been investigated so far. Since PLM, however, closely resemble the pain-evoked lower limb triple flexion response, which consists of a dorsiflexion of the ankle and flexion of the knee and hip, with an involvement of the tensor fasciae latae, hamstrings, and tibialis anterior muscles, use of a corresponding upper limb muscle might have been better suited to investigate PAM. Indeed, the triple flexion response to pain in the upper limbs consists of an adduction of the shoulder (pectoralis major, teres major, latissimus dorsi), flexion of the elbow (brachialis, brachioradialis, biceps brachii), and pronation of the arm (pronator teres, pronator quadratus). Therefore, we cannot rule out that the use of one of these upper limb muscles, rather than the flexor digitorum superficialis, might have led to a higher PAM and periodicity index.

In summary, PAM are not uncommon in healthy subjects, and occur predominantly during wakefulness with no apparent true periodicity. It might be suggested that, in contrast to classical PLM, at least some PAM do not present a true periodic phenomenon but are merely random voluntary movements meeting the wide range of periodicity criteria for PLM.

## Funding sources

This study was supported by the 10.13039/501100002428Austrian Science Fund (FWF), project no. KLI 236. The recruitment of the healthy study participants was supported by the intramural funding program of Innsbruck Medical University for young scientists, project no. 2010012005.

## Conflicts of interest

None declared.

The ICMJE Uniform Disclosure Form for Potential Conflicts of Interest associated with this article can be viewed by clicking on the following link: http://dx.doi.org/10.1016/j.sleep.2014.05.014.

Conflict of interest ICMJE Form for Disclosure of Potential Conflicts of Interest form.

## References

[bib0010] Hornyak M., Feige B., Riemann D., Voderholzer U. (2006). Periodic leg movements in sleep and periodic limb movement disorder: prevalence, clinical significance and treatment. Sleep Med Rev.

[bib0015] Freedom T., Merchut M.P. (2003). Arm restlessness as the initial symptom in restless legs syndrome. Arch Neurol.

[bib0020] Yokota T., Shiojiri T., Hirashima F. (1995). Sleep-related periodic arm movement. Sleep.

[bib0025] Wetter T.C., Brunner H., Collado-Seidel V., Trenkwalder C., Winkelmann J. (2002). Sleep and periodic limb movements in corticobasal degeneration. Sleep Med.

[bib0030] Munhoz R.P., Arruda W.O., Teive H.A. (2012). An upper limb variant of RLS? Report of 2 cases. Clin Neurol Neurosurg.

[bib0035] Ruppert E., Cretin B., Meyer C., Kilic-Huck U., Bourgin P. (2012). Characterization of periodic upper limb movement disorder in a patient with restless arms syndrome. Mov Disord.

[bib0040] Michaud M., Chabli A., Lavigne G., Montplaisir J. (2000). Arm restlessness in patients with restless legs syndrome. Mov Disord.

[bib0045] Horvath J., Landis T., Burkhard P.R. (2008). Restless arms. Lancet.

[bib0050] Chabli A., Michaud M., Montplaisir J. (2000). Periodic arm movements in patients with the restless legs syndrome. Eur Neurol.

[bib0055] Bixler E.O., Kales A., Vela-Bueno A., Jacoby J.A., Scarone S., Soldatos C.R. (1982). Nocturnal myoclonus and nocturnal myoclonic activity in the normal population. Res Commun Chem Pathol Pharmacol.

[bib0060] Ancoli-Israel S., Kripke D.F., Mason W., Kaplan O.J. (1985). Sleep apnea and periodic movements in an aging sample. J Gerontol.

[bib0065] Mosko S.S., Dickel M.J., Paul T., LaTour T., Dhillon S., Ghanim A. (1988). Sleep apnea and sleep-related periodic leg movements in community resident seniors. J Am Geriatr Soc.

[bib0070] Dickel M.J., Mosko S.S. (1990). Morbidity cut-offs for sleep apnea and periodic leg movements in predicting subjective complaints in seniors. Sleep.

[bib0075] Ancoli-Israel S., Kripke D.F., Klauber M.R., Mason W.J., Fell R., Kaplan O. (1991). Periodic limb movements in sleep in community-dwelling elderly. Sleep.

[bib0080] Pennestri M.H., Whittom S., Adam B., Petit D., Carrier J., Montplaisir J. (2006). PLMS and PLMW in healthy subjects as a function age: prevalence and interval distribution. Sleep.

[bib0085] Frauscher B., Gabelia D., Mitterling T., Biermayr M., Bregler D., Ehrmann L. (2014). Motor events during healthy sleep: a quantitative polysomnographic study. Sleep.

[bib0090] Frauscher B., Iranzo A., Gaig C., Gschliesser V., Guaita M., Raffelseder V. (2012). Normative EMG values during REM sleep for the diagnosis of REM sleep behavior disorder. Sleep.

[bib0095] Zucconi M., Ferri R., Allen R., Baier P.C., Bruni O., Chokroverty S. (2006). International Restless Legs Syndrome Study Group (IRLSSG). The official World Association of Sleep Medicine (WASM) standards for recording and scoring periodic leg movements in sleep (PLMS) and wakefulness (PLMW) developed in collaboration with a task force from the International Restless Legs Syndrome Study Group (IRLSSG). Sleep Med.

[bib0100] Ferri R., Zucconi M., Manconi M., Plazzi G., Bruno O., Ferini-Strambi L. (2006). New approaches for the study of periodic leg movments during sleep in restless legs syndrome. Sleep.

[bib0105] Provini F., Vetrugno R., Meletti S., Plazzi G., Solieri L., Lugaresi E. (2001). Motor pattern of periodic limb movements during sleep. Neurology.

